# A Flexible and Thermally Uniform TiO_2_/Ag/SiO_2_ Transparent Heater for Skin-Integrated Applications

**DOI:** 10.3390/jfb17030151

**Published:** 2026-03-18

**Authors:** Jaejeong Jo, Geonwoo Kang, Chankyoung Lee, Tran Thi Bao Vo, Dooho Choi

**Affiliations:** Department of Semiconductor Engineering, Gachon University, 1342 Seongnam-daero, Sujeong-gu, Seongnam-si 13120, Gyeonggi-do, Republic of Korea; finances@gachon.ac.kr (J.J.); kang000606@gachon.ac.kr (G.K.); tozion1@gachon.ac.kr (C.L.); baotran270196@gachon.ac.kr (T.T.B.V.)

**Keywords:** functional biomaterials, skin-integrated devices, wearable thermotherapy, transparent heaters, flexible electronics

## Abstract

Transparent heaters intended for skin-contacting applications must simultaneously satisfy optical transparency, mechanical compliance, thermal uniformity, and operational safety under biologically relevant temperature ranges. Here, we evaluate the applicability of a TiO_2_/Ag/SiO_2_ (TAS) dielectric–metal–dielectric transparent heater as a functional biomaterial platform for wearable and skin-integrated thermal systems. By systematically optimizing each layer thickness of the TAS structure, the heater achieves high visible-light transmittance (average of 86.6%) together with low sheet resistance on the order of 7.7 Ω/sq for low-voltage operation. The TAS heater demonstrates rapid and reproducible Joule-heating behavior, showing fast thermal response with short thermal time constants and spatially homogeneous temperature distributions without localized hot spots. Stable electrothermal performance is maintained under repeated on/off cycling and during cyclic mechanical bending down to small radii, confirming excellent mechanical stability under repeated bending relevant to wearable applications. Importantly, direct on-skin evaluations conducted by attaching the device to a human elbow reveal conformal contact, uniform heating at therapeutically relevant temperatures (50–70 °C), and stable operation under dynamic bending and extension. The absence of thermal inhomogeneity during motion highlights the intrinsic stability of the TAS architecture for skin-interfaced use. Given the high optical visibility, mechanical compliance, thermal uniformity, and electrothermal stability, the proposed TAS architecture represents a promising functional biomaterial platform for wearable thermotherapy, skin-mounted healthcare devices, and human-interactive thermal systems operating under continuous mechanical deformation and direct skin contact.

## 1. Introduction

Transparent heaters (THs) are emerging as key components in next-generation wearable and deformable electronic platforms, where they function either as standalone thermal elements or as integrated modules combined with other medical and healthcare devices [1,2,3,4,5]. In such systems, transparent heaters must deliver not only high optical transparency and efficient thermal output but also maintain stable operation during continuous bending, twisting, stretching, and intimate contact with soft biological surfaces. In particular, on-skin transparent heaters have increasing attention for applications such as cutaneous thermotherapy patches for pain relief, localized heating for muscle relaxation, thermal regulation for wound-healing environments and cosmetic or dermatological treatment patches that require uniform, controllable heating directly on human skin [6,7,8]. In many of these applications, transparent heaters are further integrated with sensing, imaging, or drug-delivery components [3,9,10], necessitating optical clarity that allows visual inspection or optical signal transmission through the heating layer. These systems demand heater structures capable of stable optoelectronic operation under mechanical deformation while maintaining low-voltage functionality compatible with portable or body-mounted power sources [11,12]. When transparent heaters are used directly on skin—either alone or in combination with other functional layers—additional requirements arise. The device must exhibit biomechanical compliance to conform gently to curved and dynamically moving surfaces, thermal uniformity to avoid localized hot spots, and operational safety at therapeutically relevant temperatures (e.g., up to 70 °C) to prevent irritation or thermal damage. Reliable skin-integrated operation also requires robust mechanical endurance under long-term cyclic bending, exposure to sweat or moisture, and dynamic daily activities. Satisfying this combination of optical, electrical, mechanical, and biological requirements remains challenging, as most transparent conductors inherently suffer from a trade-off between transmittance and conductivity and often undergo performance degradation under repetitive mechanical deformation [13,14]. Commercial transparent heaters are predominantly based on transparent conducting oxides (TCOs), with indium tin oxide (ITO) being the most widely used [1,15]. Although ITO offers high visible transmittance (>80%) and relatively low sheet resistance (~10 Ω/sq), its intrinsic brittleness, susceptibility to microcracking, and requirement for high-temperature processing (>250 °C) render it unsuitable for skin-mounted or deformable thermal systems [16,17]. In addition, indium is a relatively scarce and costly element, and concerns regarding long-term supply stability further limit the economic sustainability of large-area ITO-based transparent heaters [18,19]. Alternative conductors such as carbon nanotubes [20,21,22], graphene [23,24,25], metal nanowires [26,27,28,29,30] and conductive polymers [31,32] have shown promise but still face limitations including junction instability, oxidation, poor durability under sweat exposure, or surface roughness that complicates intimate skin contact.

Dielectric–metal–dielectric (DMD) multilayer architectures have recently emerged as a high-performance alternative, as they can simultaneously achieve high optical transmittance, low sheet resistance, and excellent mechanical flexibility [33,34,35]. In these structures, the ultrathin metallic layer serves as in-plane conduction pathway—distinct from the percolative network conduction found in carbon-nanotube- or metal-nanowire-based heaters—resulting in more stable and efficient Joule heating, while dielectric layers enhance optical interference, improve metal adhesion, and protect against environmental degradation. These combined attributes make DMD-based heaters particularly advantageous for wearable and skin-attachable devices that must conform to curved, soft, and dynamically moving surfaces while maintaining reliable thermal output. In this study, we propose a flexible TiO_2_/Ag/SiO_2_ (TAS) transparent heater for wearable biomedical applications. TiO_2_, known as an effective wetting promoter for noble metals [36], was employed as the bottom dielectric layer to facilitate uniform Ag nucleation and secure layer adhesion, while SiO_2_, possessing a lower refractive index of 1.48 (550 nm) [37] than the value of 2.45 (550 nm) for TiO_2_ [38], was selected as the top dielectric to maximize optical transparency [39,40] and protect the Ag layer [41,42]. Through systematic optimization of dielectric thicknesses and investigation of the Ag morphology transition, we establish an optimized DMD configuration that yields high transmittance and low sheet resistance. In addition to the fundamental optoelectrical optimization, this work integrates essential evaluations for on-skin biomaterial devices. The TAS architecture is fabricated using conventional magnetron sputtering, an industry-established and scalable deposition technique widely adopted for large-area transparent conductive coatings. The use of ultrathin layers without complex lithographic patterning further supports cost-effective and scalable production. Long-term cyclic bending tests were conducted to verify mechanical endurance under repetitive deformation, mimicking real wearable conditions. Furthermore, the heater was directly attached onto human skin to assess conformal contact, thermal uniformity across biological surfaces, and safety during dynamic bending and straightening motions. This demonstrates not only stable thermal outputs but also the capability to visually monitor skin appearance during heating, positioning it as a promising functional biomaterial platform for next-generation wearable therapeutic devices, conformal healthcare electronics, and human-interactive thermal systems.

## 2. Experiments

TAS-THs were fabricated on a flexible polyimide (PI) substrate via a multi-target magnetron sputtering system, using 99.99 wt% SiO_2_ (3-inch), TiO_2_ (2-inch) and 99.999 wt% Ag (2-inch) targets. The chamber base pressure was maintained below 2.67 × 10^−4^ Pa, and all depositions were performed in an Ar ambient at 0.27 Pa. Prior to deposition, each target was pre-sputtered to remove surface contamination. RF powers were set to 70 W for TiO_2_, 70 W for SiO_2_ and 50 W for Ag. Deposition rates, calibrated using thick (>200 nm) films measured by a surface profilometer (D-100, KLA, Milpitas, CA, USA) were 0.49, 0.43 and 4.58 Å/s for TiO_2_, SiO_2_ and Ag, respectively. The substrates were rotated continuously at 8.5 rpm during deposition to ensure uniform film growth. All layers were deposited in situ without exposure to air. Following TAS stack formation, Ag electrodes (5 mm wide, 100 nm thick) were deposited along parallel edges via RF sputtering for electrical contact.

The sheet resistance of the fabricated films was measured using a four-point probe system (CMT-100S, AIT, Suwon, Republic of Korea) at five randomly selected locations per sample, and the average value was reported. Optical transmittance spectra in the 400–800 nm range were acquired with a spectrophotometer (CARY-100, Agilent, Santa Clara, CA, USA). The surface morphology of the Ag layer was examined by field-emission scanning electron microscopy (FE-SEM, JSM-IT800, JEOL, Tokyo, Japan; DEU Converging Materials Core Facility, Busan, Republic of Korea, NFEC-2021-12-275266); for this purpose, the SiO_2_ overlayers were omitted to allow direct observation of the Ag surface structure. Joule heating performance was evaluated by applying direct current through the device using a DC power supply (EPS-3303, EZT, Seoul, Republic of Korea), while real-time thermal profiles were monitored using an infrared (IR) thermal imager (TrueIR U5857A, Keysight Technologies, Santa Rosa, CA, USA). Mechanical flexibility was tested using a bending tester (STM-2-TBDS, Sciencetown, Seoul, Republic of Korea) under various bending radii, during which the surface temperature was measured to evaluate thermal variations under mechanical deformation. The accelerated humidity test (85 °C/85% RH) was conducted using a temperature–humidity chamber (TH-GA-180, JEIO TECH, Daejeon, Republic of Korea).

To evaluate the feasibility of the transparent heater as a skin-contacting functional biomaterial, on-skin heating tests were conducted by directly attaching the device to the human elbow region, which undergoes repeated bending and extension during daily motion. The heater was gently laminated onto the skin, ensuring intimate conformal contact without introducing external mechanical strain to the device. Joule heating was activated under controlled DC bias while the elbow was sequentially maintained in both relaxed and naturally bent states. Real-time surface temperature distributions were monitored using an infrared (IR) thermal imaging camera, from which standard deviation values were extracted from the IR images after excluding regions within 5 mm of the device edges. For the images for the bending radii of 4 and 6 mm, the central 40% area was selected for standard deviation analysis. Heating performance was examined under stepwise temperature modulation within therapeutically relevant ranges (50–70 °C) to assess thermal uniformity and controllability. Optical photographs and corresponding IR images were recorded at each temperature and deformation state, confirming uniform heat distribution and reliable operation under dynamic skin motion.

## 3. Results and Discussion

**Figure 1:**
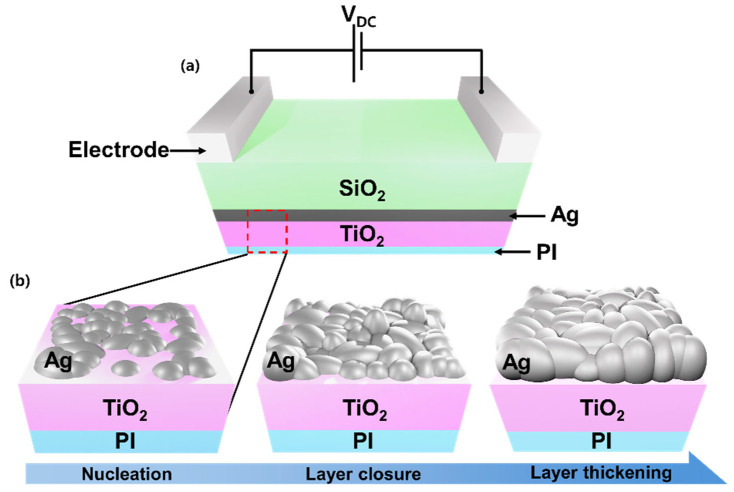
(**a**) Schematic illustration of a TiO_2_/Ag/SiO_2_ (TAS) transparent heater fabricated on a flexible PI substrate. (**b**) Morphological evolution of the Ag layers—illustrating island nucleation, film closure and subsequent post-closure thickening.

Figure 1 presents the structural design and growth concept of the flexible TiO_2_/Ag/SiO_2_ (TAS) transparent heater fabricated on a polyimide (PI) substrate. The device adopts a dielectric/metal/dielectric (DMD) configuration, in which an ultrathin Ag layer is embedded between a bottom TiO_2_ layer and a top SiO_2_ layer to simultaneously satisfy optical transparency, electrical efficiency, and mechanical compliance required for skin-integrated operation. The bottom TiO_2_ layer plays a dual role in the TAS architecture. Owing to its high surface energy and strong metal–oxide interaction, TiO_2_ promotes uniform Ag nucleation and accelerates the formation of a continuous metallic film at reduced thickness [36], which is critical for achieving stable electrical conduction at low operating voltages. At the same time, TiO_2_ functions as an optical interference layer, contributing to enhanced visible transmittance within the multilayer stack [34,43]. The top dielectric layer consists of low-refractive-index SiO_2_, which suppresses Fresnel reflection at the air/oxide interface and further improves optical transparency [39,44]. In addition, the SiO_2_ overlayer serves as a protective barrier against oxidation and environmental degradation of the Ag film, thereby supporting long-term operational stability during skin contact and exposure to ambient conditions. The ultrathin Ag layer acts as the primary current-carrying and heat-generating component [45,46,47]. When a DC bias is applied across the parallel electrodes shown in Figure 1a, electrical current flows laterally through the Ag layer, producing resistive Joule heating. Owing to the planar geometry and homogeneous current distribution, the TAS heater enables rapid and spatially uniform temperature rise, which is essential for safe and comfortable skin-interfaced biomedical applications. Figure 1b schematically illustrates the morphological evolution of the Ag layer as a function of thickness. At the initial deposition stage, Ag grows in the form of isolated islands on the TiO_2_ surface (nucleation stage). With increasing thickness, these islands enlarge and coalesce, forming continuous electrical conduction pathways (layer-closure stage). Beyond the closure thickness, the continuous Ag films undergo further thickening (film-thickening stage). This morphological transition critically governs the functional performance of the transparent heater. Discontinuous Ag layers result in significant reduction in electrical conduction, elevated operating voltages and spatially nonuniform heating, which are undesirable for wearable and body-mounted systems. Moreover, nanoscale discontinuities introduce optical inhomogeneity and localized light–matter interactions [48], degrading transparency and hindering visual monitoring of the underlying skin. Incomplete metal coverage also compromises interfacial integrity [49], increasing susceptibility to mechanical degradation under repeated bending and dynamic skin motion. Once a continuous Ag film is established, stable electrical pathways enable low-voltage Joule heating and spatially homogeneous temperature distribution, both of which are indispensable for safe skin contact. Although further increases in Ag thickness reduce electrical resistance and enhance heating efficiency, excessive metal thickness leads to increased optical absorption, limiting transparency and visual access to the skin. Therefore, precise control of Ag film growth and closure behavior is essential to balance electrical efficiency, optical visibility, and mechanical robustness—key parameters that collectively define the practical suitability of transparent heaters as functional biomaterials for wearable and skin-interfaced biomedical applications.

**Figure 2:**
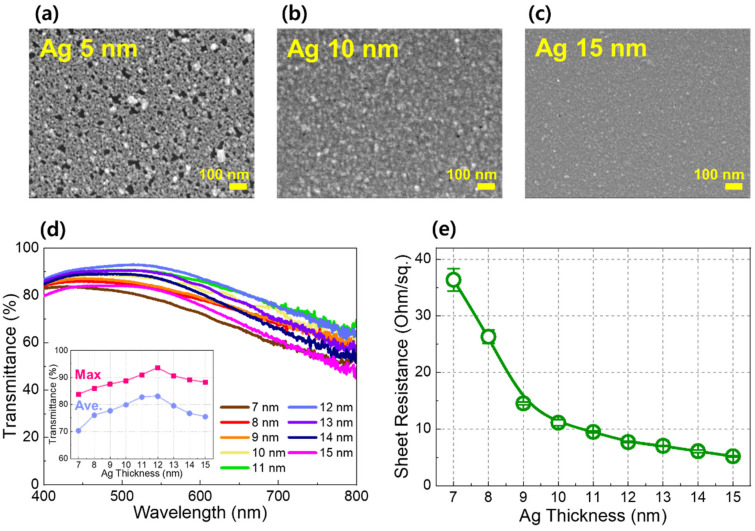
(**a**–**c**) SEM images of the Ag layer in the TAS heater structure at Ag thicknesses of 5, 10 and 15 nm, respectively. To observe the Ag surface morphology, the top SiO_2_ layer was intentionally omitted. (**d**) Visible light (λ: 400–800 nm) transmittance of the TAS heater as a function of Ag thickness ranging from 7 nm to 15 nm, with a 1 nm increment. The inset summarizes the maximum and average transmittance as a function of Ag layer thickness. All transmittance spectra were measured using an PI substrate as the baseline. (**e**) Sheet resistance of the TAS heater as a function of Ag thickness ranging from 6 nm to 15 nm.

Figure 2 shows the thickness-dependent structural, optical, and electrical characteristics of the Ag layer in the TAS structure. Figure 2a–c present SEM images of the Ag layer deposited with thicknesses of 5, 10, and 15 nm, respectively, illustrating the morphological evolution as a function of Ag thickness. At a thickness of 5 nm, the Ag layer consists of discontinuous island-like structures, indicating incomplete surface coverage. As the Ag thickness increases, the islands grow laterally and coalesce, and a laterally continuous thin film is clearly formed at thicknesses of 10 nm and above, signifying the completion of Ag layer closure. This thickness-dependent evolution is consistent with a Volmer–Weber-type growth behavior, which is commonly observed for high-surface-energy noble metal films deposited on low-surface-energy oxide surfaces [34,50,51]. Figure 2d shows the visible-light transmittance of the TAS structure as a function of Ag thickness, measured while fixing the thicknesses of the bottom TiO_2_ and top SiO_2_ layers at 20 and 60 nm, respectively, to isolate the thickness effect of the Ag layer. The transmittance exhibits a non-monotonic dependence on Ag thickness, reflecting the combined influence of Ag film morphology and optical interference within the dielectric/metal/dielectric stack. Notably, the maximum transmittance is observed at a Ag thickness of approximately 12 nm, which is close to the thickness regime where the Ag layer transitions into a continuous film. At lower thicknesses, discontinuous Ag films exhibit reduced transmittance due to enhanced localized surface plasmon resonance (LSPR) and scattering losses associated with isolated metal islands. In contrast, at higher thicknesses beyond the closure regime, increased optical absorption in the Ag layer leads to a gradual decrease in transmittance. This interplay results in an optimal Ag thickness near the film-closure threshold, where both morphological continuity and optical transparency are favorably balanced. Figure 2e presents the corresponding sheet resistance as a function of Ag thickness. A pronounced decrease in sheet resistance is observed as the Ag thickness approaches and exceeds the layer-closure regime, indicating the formation of continuous electrical conduction pathways.

**Figure 3:**
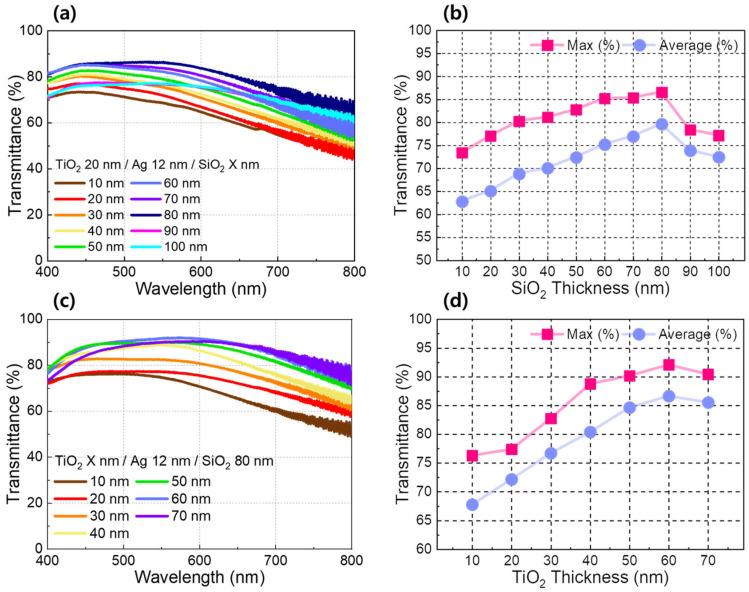
(**a**) Visible light transmittance spectra of the TAS heater with the top SiO_2_ layer thickness ranging from 10 nm to 100 nm in 10 nm increments. (**b**) corresponding maximum and average transmittance values extracted from (**a**) as a function of SiO_2_ layer thickness. (**c**) Visible light transmittance spectra with the bottom TiO_2_ layer thickness ranging from 10 nm to 70 nm in 10 nm increments. (**d**) Corresponding maximum and average transmittance values extracted from (**c**) as a function of TiO_2_ layer thickness. All transmittance spectra were measured using an PI substrate as the baseline.

Figure 3 examines the optical characteristics of the TAS heater through a stepwise optimization of the dielectric layer thicknesses, with the Ag layer thickness fixed at 12 nm based on the results in Figure 2. All transmittance measurements were performed using a PI baseline reference. In the first step, the thickness of the top SiO_2_ layer was systematically varied to identify the condition yielding the highest optical transparency. Figure 3a,b present the visible-light transmittance spectra and the corresponding maximum and average transmittance values as the SiO_2_ thickness was increased from 10 to 110 nm, while the bottom TiO_2_ layer thickness was kept constant at 20 nm. As the SiO_2_ thickness increases, the average visible transmittance improves markedly, reaching a maximum value of 86.6% at a thickness of 80 nm. Further increases beyond this point lead to a decline in transmittance. This trend indicates the presence of an optimal SiO_2_ thickness under the given conditions. The observed behavior reflects the anti-reflective function of the SiO_2_ overlayer, whose thickness-dependent optical interference modulates surface reflection and light transmission through the TAS stack. After fixing the top SiO_2_ thickness at the optimized value identified above, the thickness of the bottom TiO_2_ layer was subsequently varied to further refine the optical response of the TAS heater. Figure 3c,d show the visible-light transmittance spectra and extracted optical metrics as the TiO_2_ thickness was increased over a range of 10–70 nm, while maintaining the Ag and SiO_2_ thicknesses at 12 and 80 nm, respectively. Similar to the observation in Figure 3a,b, thickness variations of TiO_2_ layers strongly influence the optical interference condition within the DMD architecture. As a result, the highest average and maximum visible transmittance were reached at 60 nm. In summary, the sequential optimization of the top SiO_2_ and bottom TiO_2_ layers enables effective control over visible-light transmission in the TAS heater, while preserving the Ag thickness required for stable electrical conduction. Such an optically optimized configuration is particularly advantageous for transparent heater applications that demand high visibility in conjunction with uniform thermal performance, including wearable and skin-contacting biomedical devices. To further clarify the relative performance of the proposed TAS heater, a quantitative comparison with representative transparent heater technologies (ITO, graphene, carbon nanotube and metal nanowires) is summarized in Table 1. The comparison includes optical transmittance, sheet resistance, and key structural characteristics reported in prior studies. These results indicate that the TAS heater exhibits a balanced combination of high optical transmittance and low sheet resistance, together with structural stability and mechanical flexibility, as will be further discussed in the following sections.

**Figure 4:**
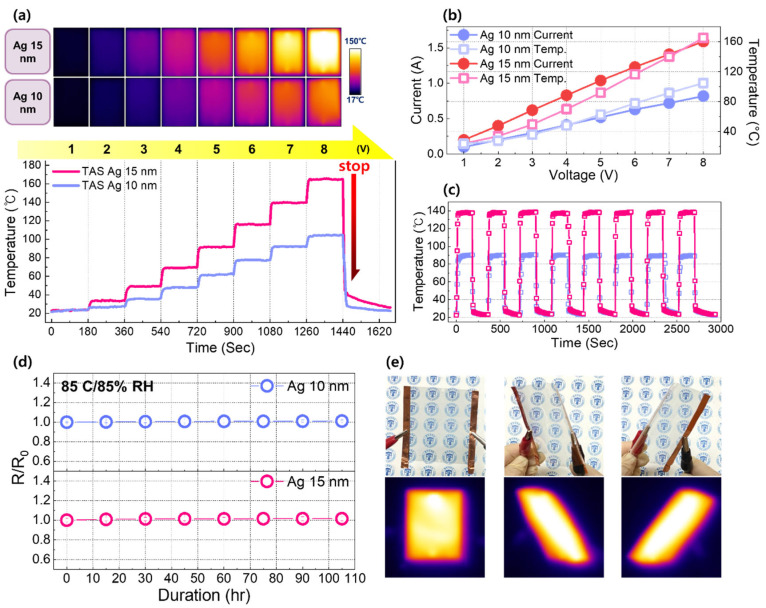
Joule heating performance of the TAS heaters with Ag thicknesses of 10 nm and 15 nm. (**a**) Time-resolved temperature profiles under stepwise increase in the applied voltage. IR images captured at each applied voltage for both devices. For the 15 nm Ag sample, the standard deviations for the thermal distributions while applying voltages from 1 V to 8 V were 0.4, 1.0, 2.1, 3.0, 4.6, 6.8, 9.8, and 8.7 °C, respectively, whereas for the 10 nm Ag sample, the corresponding values were 0.4, 0.8, 1.2, 1.6, 3.4, 4.4, 5.1, and 6.9 °C, respectively. (**b**) Corresponding I-V and T-V characteristics as a function of applied bias, highlighting the correlation between electrical input and thermal output for the two heaters. (**c**) Repeated on/off cycling test conducted under a fixed bias with 180 s on/off intervals, demonstrating the reproducibility and operational stability of the TAS heaters. The pink and blue curves correspond to the 15 nm and 10 nm Ag heaters, respectively. (**d**) Time-dependent variation of normalized sheet resistance of the optimized TAS heater during accelerated humidity testing at 85 °C/85% RH for 72 h. The resistance variation remained within 1.5%, indicating stable electrical performance under severe moisture exposure conditions. (**e**) Photographs of a TAS heater in flat and deformed configurations, placed over a logo, along with the corresponding IR thermal images captured during operation.

Figure 4 evaluates the Joule-heating behavior of TAS heaters incorporating Ag thicknesses of 10 and 15 nm, which possess a pronounced difference in the sheet resistance (11.1 and 5.19 Ω/sq respectively). A series of DC voltages was sequentially applied up to 8 V in 1 V increments, with each voltage step maintained for 180 s, while the real-time surface temperature was continuously monitored. As shown in Figure 4a, both heaters exhibit stable and monotonic temperature increases with increasing applied voltage, consistent with Joule’s law governing resistive heat generation in conductive films. Notably, each voltage increment induces a rapid temperature rise followed by prompt thermal stabilization, indicating a short thermal time constant and minimal thermal inertia. Such fast and stable thermal responses are particularly desirable for skin-contacting thermal devices, where precise and controllable temperature modulation is critical. At all applied voltage levels, the heater incorporating a 15 nm Ag layer consistently reaches higher surface temperatures than its 10 nm counterpart. This behavior originates from the nonlinear nature of Joule heating (P = I^2^R), in which the increase in current associated with reduced sheet resistance outweighs the simultaneous decrease in resistance. As a result, the thicker Ag layer enables higher power dissipation and enhanced heating efficiency under the same bias conditions. Importantly, both devices exhibit rapid thermal responses, reaching 90% of target temperatures within 10 s. Further, the accompanying infrared (IR) thermal images reveal laterally uniform temperature distributions without localized hot spots, confirming homogeneous heat generation across the active area—an essential requirement for safe and comfortable contact with human skin. Figure 4b summarizes the heater current and temperatures as a function of the applied voltage. The current–voltage (I-V) characteristics exhibit linear Ohmic behavior over the measured voltage range. The temperature–voltage (T-V) relationship exhibits a superlinear dependence, reflecting the quadratic relationship between Joule heating and current (T is proportional to I^2^R). This divergence highlights the coupled electronic and thermal transport processes that govern the heating behavior of the TAS heaters. Figure 4c presents the heating–cooling cycling behavior under a constant bias with 180 s on/off intervals. The thermal response remains fully reversible and stable over repeated cycles, with no noticeable degradation in peak temperature, heating rate, or cooling behavior. To further evaluate environmental stability, accelerated humidity testing was performed at 85 °C/85% RH for 105 h. As shown in Figure 4d, the normalized sheet resistance remained within 1.5% variation throughout the test, indicating stable electrical performance under severe moisture exposure. In addition, the heating performance of the optimized TAS heater was examined in both flat and deformed configurations, as shown in Figure 4e. The device maintained stable and spatially uniform temperature distributions without noticeable hot spots or performance degradation under deformation. A more systematic evaluation of the flexible characteristics is presented in the following section. These reproducible and stable thermal operations demonstrate excellent reliability and durability, supporting the suitability of TAS heaters for flexible, wearable, and skin-interfaced biofunctional heating applications.

**Figure 5:**
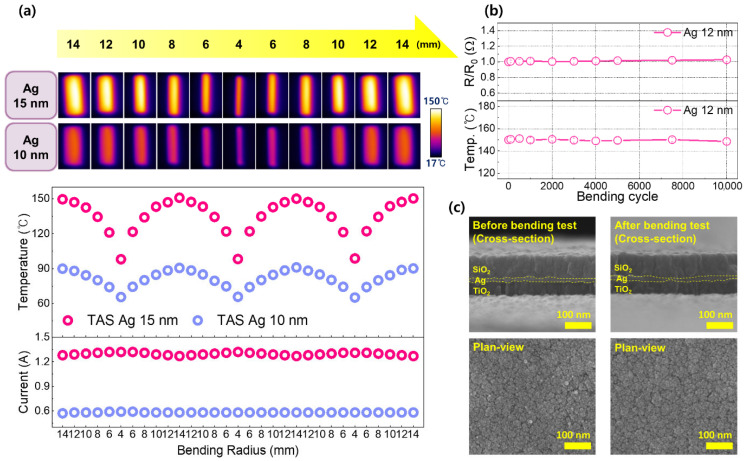
(**a**) Measured temperature and current of TAS heaters incorporating Ag layers with thicknesses of 10 and 15 nm as functions of bending radius, varied in the range of 14–4 mm. For the 15 nm Ag sample, the standard deviations of the thermal images measured at radii from 14 to 4 mm were 6.6, 7.5, 8.4, 8.4, 8.4, and 5.9 °C, respectively. At the same radii, for the 10 nm Ag sample, the standard deviations were 3.7, 3.8, 3.7, 3.5, 3.9, and 3.2 °C, respectively. From the thermal images acquired in the flat and bent states, the standard deviations of the temperature distribution were 6.1 and 8.5 °C, respectively. (**b**) Normalized resistance (R/R_0_) and operating temperature of the optically optimized TAS heater as functions of repeated bending cycles between radii of 14 and 4 mm, showing negligible (<0.5%) degradation in electrical and thermal performance. Temperature and resistance were measured at a bending radius of 4 mm. (**c**) Cross-sectional and plan-view SEM images of the optically optimized TAS heater before and after 100,000 bending cycles at a 4 mm bending radius, showing no discernible structural degradation, crack formation, or interfacial delamination, thereby validating the mechanical stability of the TAS architecture.

Figure 5 systematically evaluates the mechanical durability and thermal reliability of the TAS transparent heater under repeated cyclic bending. As shown in Figure 5a, bending tests were performed by sequentially decreasing the bending radius from 14 mm to 4 mm and subsequently increasing it back to 14 mm, thereby subjecting the device to repeated bending deformation. This bending cycle was applied to TAS heaters incorporating Ag layers with thicknesses of 10 and 15 nm to examine the influence of cyclic bending on electrothermal performance. During the bending cycles, both the surface temperature and operating current gradually decreased as the bending radius was reduced from 14 mm to 4 mm, and subsequently recovered as the radius was restored to 14 mm, resulting in a highly symmetric and reproducible response. The complete recovery of both parameters upon reverse bending indicates that the observed variations are fully reversible and do not originate from mechanical damage or degradation of conductive pathways, confirming the mechanical stability under repeated bending of the TAS multilayer structure. The transient temperature reduction at smaller bending radii is attributed to enhanced heat dissipation under bent configurations, where increased curvature results in larger exposed surface area and thus promote convective heat loss to the surrounding environment. Across all bending cycles, heaters incorporating a 15 nm-thick Ag layer consistently exhibited higher surface temperatures and operating currents, consistent with the results in Figure 4. The absence of localized hot spots in the IR images in Figure 5a indicates that cyclic bending does not induce localized resistance modulation or current crowding. Such spatially homogeneous heating under repeated bending deformation is essential for flexible heater applications that require reliable and stable operation under continuous mechanical motion. To systematically evaluate the long-term heater durability under repeated mechanical deformation, cyclic bending between radii of 14 and 4 mm was applied for up to 10,000 cycles while maintaining the heater temperature at 150 °C as shown in Figure 5b. For this testing, the optically optimized structure of TiO_2_ (60 nm)/Ag (12 nm)/SiO_2_ (80 nm) was selected. Throughout the bending cycles, the normalized resistance (R/R_0_) and operating temperature remain nearly constant, exhibiting variations below 2%. Following the cyclic bending test, cross-sectional and plan-view SEM images were obtained both before and after 100,000 bending cycles at a bending radius of 4 mm, as shown in Figure 5c. A direct comparison of the images reveals no discernible differences in layer morphology, and no evidence of crack formation, interfacial delamination, or structural discontinuity is observed after prolonged cyclic deformation. These observations are consistent with previous reports indicating that irreversible electrothermal degradation in transparent heaters is typically accompanied by crack formation or film discontinuity in the conductive layer [3,15,63]. Considering that the test temperature (150 °C) and deformation condition (4 mm bending radius) are substantially more severe than typical skin-contact conditions, the preserved structural integrity further supports the mechanical robustness and electrothermal stability of the TAS architecture for flexible skin-integrated transparent heater applications.

**Figure 6:**
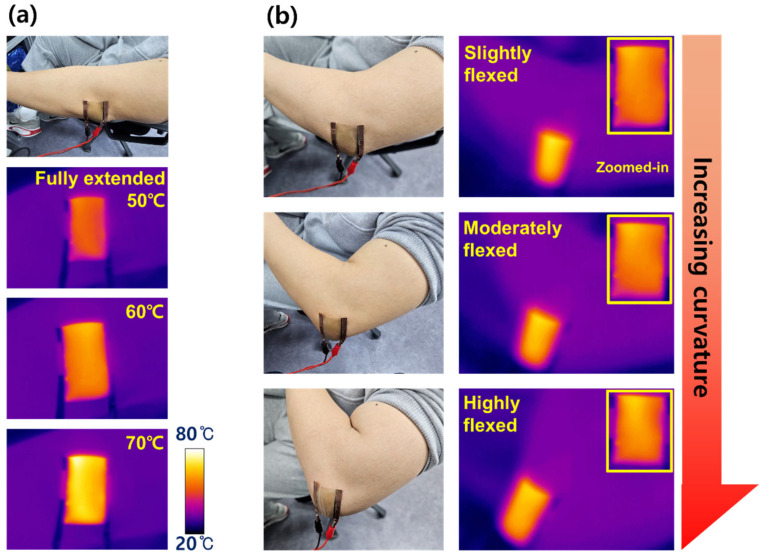
(**a**) Optical photographs and corresponding IR images of the TAS transparent heater attached to a human elbow in the fully extended state, operated at 50, 60, and 70 °C. (**b**) Optical photographs and corresponding IR images of the TAS heater operated at 60 °C under different elbow configurations, including slightly flexed, moderately flexed, and highly flexed states. Zoomed-in IR images showing the uniform heating behavior of the device, demonstrating stable thermal performance under progressive mechanical deformation. The standard deviations extracted from the thermal images in (**a**) were 3.4, 2.1, and 2.8 °C, respectively, and those in Figure 6b were 2.1, 2.7, and 3.6 °C, respectively.

Figure 6 demonstrates the on-skin heating performance and mechanical adaptability of the TAS transparent heater when directly attached to a human elbow, which undergoes dynamic curvature changes during natural motion. In the present study, the device was fabricated on a PI substrate, which is widely regarded as chemically stable and non-toxic for flexible electronic applications; however, in practical implementations, other biocompatible and stretchable substrates capable of supporting continuous thin-film growth may also be employed. As shown in Figure 6a, the heater operates in the fully extended state at target temperatures of 50, 60, and 70 °C, while the corresponding infrared (IR) thermal images confirm a uniform temperature distribution over the entire active area. No localized hot spots or temperature gradients are observed, indicating stable Joule heat generation and effective thermal spreading under direct skin contact. This behavior is essential for wearable thermal devices, where controllable and spatially uniform heating directly impacts user safety and comfort. Figure 6b further evaluates the thermal stability of the TAS heater under different elbow configurations, including slightly flexed, moderately flexed, and highly flexed states, while maintaining a constant operating temperature of 60 °C. Despite the progressive increase in bending curvature, the IR images—including the enlarged views—consistently show sufficiently homogeneous temperature profiles. The extracted standard deviation values provided in the caption of Figure 6 correspond to relative temperature deviations well below 10% of the operating temperature range, quantitatively confirming the absence of localized hot spots and the laterally uniform Joule-heating behavior of the TAS heater. The absence of strong thermal fluctuation or spatial inhomogeneity suggests that mechanical deformation does not induce localized resistance modulation or current crowding in the Ag layer. Instead, the electrothermal response remains inherently stabilized, assisted by intrinsic Joule heating feedback, through which small deformation-induced resistance variations are naturally compensated by corresponding adjustments in current flow and heat generation. Such heating characteristics highlight the capability of the TAS transparent heater to deliver stable, uniform, and self-regulated heating directly on a dynamically moving human joint, without degradation in thermal performance. This on-skin demonstration, combined with the mechanical durability and electrothermal reliability discussed earlier, underscores the practical readiness of the TAS architecture for wearable and skin-integrated transparent heater applications that demand both mechanical compliance and thermally safe operation under continuous human motion.

## 4. Conclusions

In this study, we evaluated the applicability of a TiO_2_/Ag/SiO_2_ (TAS) transparent heater as a functional biomaterial platform for wearable and skin-integrated thermal applications. The optical transmittance of the TAS structure was found to depend strongly on the thicknesses of the top and bottom dielectric layers, serving as antireflection layers. By experimentally optimizing the dielectric layer thicknesses, high optical transparency was achieved while maintaining stable electrical conduction through the ultrathin Ag layer, resulting in a low sheet resistance of 7.7 Ω/sq and an average visible-light transmittance of 86.6%. The TiO_2_ bottom layer supports uniform Ag nucleation and stable electrical pathways, whereas the SiO_2_ top layer improves optical transmission and provides environmental protection. The electrothermal performance of the TAS heater was confirmed through Joule-heating measurements, which revealed rapid and reproducible thermal responses, reaching more than 90% of the target temperature within 10 s and maintaining stable operation during repeated on/off cycling. Mechanical reliability tests further demonstrate that both electrical resistance and operating temperature remain stable under cyclic bending down to a 4 mm radius and during extended bending up to 10,000 cycles. The suitability of the TAS heater for direct skin contact was further validated through on-skin evaluations. When attached to a human elbow, the heater maintains conformal contact and delivers uniform heating at therapeutically relevant temperatures during dynamic bending and extension. The stable thermal behavior under these conditions is supported by intrinsic Joule heating feedback, through which small resistance variations induced by mechanical deformation are passively compensated by corresponding adjustments in current and heat generation, contributing to predictable and safe heating behavior during natural human motion. Overall, this work confirms that the TAS transparent heater satisfies the key requirements of a functional biomaterial for skin-integrated thermal systems, including optically optimized transparency, mechanical compliance, thermal uniformity, and electrothermal stability under dynamic deformation. These results support the applicability of the TAS architecture for wearable thermotherapy, skin-mounted healthcare devices, and human-interactive thermal platforms that operate under continuous mechanical motion and direct skin contact.

## Figures and Tables

**Table 1:** Performance comparison of various types of transparent heaters.

Heater Type	Transmittance (%)	Sheet Resistance (Ω/Sq.)	Note	Ref.
ITO	88–90	300–2500	Non-flexible	[52,53]
ZnO	90	11–114	Non-flexible	[54,55]
Graphene	70–89	43–520	-	[24,56,57]
Carbon Nanotube	83–95	580–2600	-	[58,59,60]
Metal Nanowires	90	10–33	Low Structural Stability	[61,62]
TAS (This study)	86.6	7.7	-	-

## Data Availability

The raw data supporting the conclusions of this article will be made available by the authors on request.
